# Urgent-start peritoneal dialysis

**DOI:** 10.1590/2175-8239-JBN-2022-E009en

**Published:** 2022-10-28

**Authors:** Caio Pellizzari, Thyago Proença de Moraes

**Affiliations:** 1 Santa Casa de Misericórdia de Curitiba, Curitiba, PR, Brasil; 2 Pontifícia Universidade Católica do Paraná, Programa de Pós-Graduação em Ciências da Saúde, Curitiba, PR, Brasil

**Figure 1:**
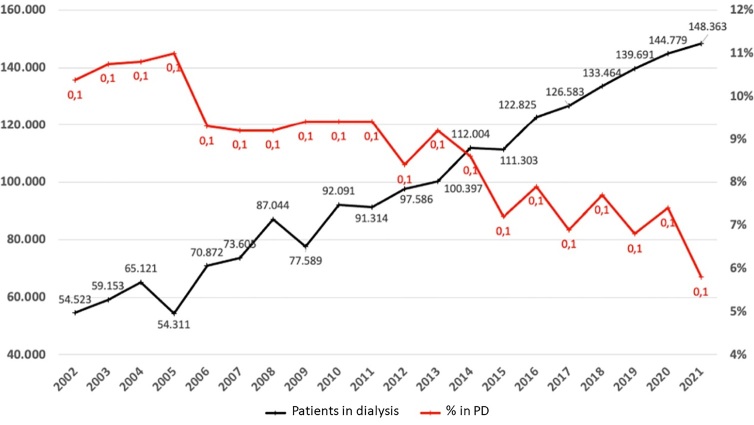
Growth of the penetration rate of peritoneal dialysis in Brazil in the last 20 years

Source: Census surveys of the Brazilian Society of Nephrology

The global prevalence of chronic kidney disease has increased significantly as a consequence of factors such as aging of the population, lifestyle changes, and improved treatment of comorbid conditions often seen in individuals with CKD and associated with cardiovascular events in general. The census surveys organized by the Brazilian Society of Nephrology since the late 1990s have shown a constant and linear increase in the number of patients prescribed renal replacement therapy, with the number of patients on dialysis growing by more than 170% in 20 years (Figure 1)^1-2^.

Hemodialysis and peritoneal dialysis (PD) are equivalent modes of RRT in terms of patient survival and metabolic control^3^. Decades ago, chronic PD was customarily initiated with the implantation of a catheter after giving the abdominal cavity a rest. Such approach is still used in some clinics for fear of complications tied to an earlier start of PD. Indications changed with the few adverse events described in the first studies about high volume PD in the treatment of acute kidney injury. Likewise, urgent-start peritoneal dialysis has been proposed for the treatment of patients with CKD as an alternative to address the growing number of incident patients on dialysis and a means to alleviate the burden of an overloaded healthcare system and prevent the exposure of patients to temporary hemodialysis vascular catheters.

In this issue of the *Brazilian Journal of Nephrology*, Pilatti et al. compared the different clinical outcomes observed during the first year of therapy of 137 patients on PD, 70 of whom prescribed urgent-start peritoneal dialysis^4^. The comparison of intermediary outcomes - such as hospitalization, treatment failure, mechanical complications, and infection associated with PD - and hard outcomes - including death - corroborated current literature findings that praised urgent-start PD for its safety. No statistically significant difference was found between any of these outcomes when therapy was initiated seven days after the implantation of a catheter or earlier. Nonetheless, matters related to the definition of what constitutes urgent-start PD and the methods used in the study in question beg discussion.

Pilatti et al. defined urgent-start PD as chronic therapy initiated within seven days of the placement of a Tenckhoff catheter, an important change from the early studies in which the time reference was 15 days. Interestingly, some centers have opted to start therapy earlier (within seven days of catheter placement) without necessarily calling it urgent-start PD. Descriptions of high-volume PD as safe for patients (particularly in Brazil) have contributed to such trend^5^. Therefore, PD initiated within less than seven days of catheter implantation might be a more adequate definition of urgent-start PD. In order to facilitate discussion, an international movement is attempting to standardize the definitions of the main events in nephrology. Urgent-start is likely to be defined as therapy initiated within 72 hours of catheter implantations^4^. Adjustments in definitions are a common event in science, and do not affect the relevance of the findings published by Pilatti et al. Although reporting on a retrospective single-center study, their article met the traditional standards of reporting survival analyses adjusted for potential confounding factors, as seen in prior publications.

There is little doubt about the importance of PD in emergency care or the benefits it may yield in the future. However, despite the attention given to urgent-start PD in the literature, barriers to its implementation in care centers still exist, including the dissemination of knowledge and data to the nephrology community, the logistics behind having nephrologists in charge of implanting catheters, and the administrative processes involving healthcare institutions concerning the authorization and payment for high complexity procedures.
